# The Small Glutathione Peroxidase Mimic 5P May Represent a New Strategy for the Treatment of Liver Cancer

**DOI:** 10.3390/molecules22091495

**Published:** 2017-09-08

**Authors:** Juxin Yin, Bingmei Wang, Xuejun Zhu, Xiaonan Qu, Yi Huang, Shaowu Lv, Ying Mu, Guimin Luo

**Affiliations:** 1 Key Laboratory for Molecular Enzymology and Engineering of the Ministry of Education, College of Life Science, Jilin University, Changchun 130000, China; yinjuxin@163.com (J.Y.); wangbingmei@163.com (B.W.); zhuxuejun@163.com (X.Z.); quxn1077@163.com (X.Q.); huangyimzd@126.com (Y.H.); muying@zju.edu.cn (Y.M.); gmluo@163.com (G.L.); 2 Research Center for Analytical Instrumentation, Institute of Cyber-Systems and Control, State Key Laboratory of Industrial Control Technology, Zhejiang University, Hangzhou 310000, China

**Keywords:** glutathione peroxidase mimic, selenium-containing peptide, tumor, liver cancer, immunity

## Abstract

Glutathione peroxidase (GPx) is an antioxidant protein containing selenium. Owing to the limitations of native GPx, considerable efforts have been made to develop GPx mimics. Here, a short 5-mer peptides (5P) was synthesized and characterized using matrix-assisted laser desorption ionization time-of-flight mass spectrometry. Enzyme coupled assays were used to evaluate GPx activity. The cell viability and apoptosis of H22 cells were tested, and mice bearing H22 cell-derived tumors were used to determine the effects of 5P on tumor inhibition. In comparison with other enzyme models, 5P provided a suitable substrate with proper catalytic site positions, resulting in enhanced catalytic activity. In our mouse model, 5P showed excellent inhibition of tumor growth and improved immunity. In summary, our findings demonstrated the design and synthesis of the small 5P molecule, which inhibited tumor growth and improved immunity. Notably, 5P could inhibit tumor growth without affecting normal growth. Based on these advantages, the novel mimic may have several clinical applications.

## 1. Introduction

Oxidative stress occurs when there is an imbalance between oxidation and anti-oxidation. Reactive oxidative species (ROS) are harmful to cells when oxidative metabolism products increase or antioxidative defenses are lacking in an organism [1,2]. Many studies have shown that ROS are related to some physiological and pathological processes and play important roles in many diseases.

Glutathione peroxidase (GPx) is an important antioxidant enzyme that was first discovered by Mills in 1957 as a selenium-containing enzyme [3]. In 1973, Flohe and Rotruck established a connection between GPx and selenium [4]. Although the structure of GPx and the biologic effects of selenium have been fully elucidated, some details of the effects of GPx still need to be clarified. Owing to the limitations associated with native GPx, including its instability, high cost, limited availability, high molecular weight, and immunogenicity [5], many scientists have attempted to study and develop GPx mimics. However, early GPx mimics have shown low activity because of the lack of a glutathione (GSH) binding site. For example, the activity of the GPx mimic ebselen is only 0.99 U/μmol [6]. Wilson introduced quaternary ammonium salt near the diselenide bridge of a GPx mimic to improve the GSH binding ability [7]. Additionally, quaternary ammonium salt can provide protons for reduction of the substrate H_2_O_2_. Thus, the activity of this type of GPx mimic (i.e., a diselenide compound) can be increased. Hilvert transformed subtilisin into a GPx mimic by chemical mutation [8], and the catalytic efficiency of the mimic was 7 × 10^4^ times more than that of diphenyl diselenide compounds when the favored 3-carboxy-4-nitrobenzenethiol was used as the substrate. However, its activity towards GSH, the substrate of native GPx, was rather low. These findings demonstrated that the key to developing GPx mimics is to generate a specific binding site for GSH [9]. The main methods for synthesis of selenoprotein are chemical synthesis and chemical modification. Chemical synthesis involves the direct insertion of selenium cysteine (Sec) into the selenium protein or peptide and is therefore suitable for use in relatively low-molecular-weight peptides. The chemical modification method mainly includes modification of chemical functional groups and recombinant fragments and involves a large amount of heat, resulting in instability of the protein structure or major degeneration. Accordingly, appropriate environmental conditions are needed.

The active site of GPx has two marked characteristics: (1) recognition of and combination with the substrate (GSH) site; and (2) the unique catalytic functional group of the Sec. Therefore, both of these factors should be considered when designing our model. According to Iwaoka, the individual amino acids have different effects on GPx-like activity [10]. However, their roles in catalytic function in terms of interactions with the Se active center have not been clarified. Additionally, Epp et al. [11] indicated that Arg-40, Gln-130 and Arg-167, which presumably form salt bridges and hydrogen bonds with the glutathione molecule, are functionally important. Accordingly, we can conclude that the GPx catalytic functional group is the Sec and that the binding site of the substrate (GSH) is made up of two arginines (forming salt bridge) and one glutamine (forming the hydrogen bond).

Here, we designed a 5-mer peptide (Arg-Gly-Sec-Arg-Asn; 5P) that contained two Arg and one Sec and used Asn instead of Gln, with introduction of Gly to improve the flexibility of the mimic. This 5P was synthesized directly by solid phase peptide synthesis instead of chemical mutation, and the analog catalytic site of the analog and synergy of binding sites were considered. Additionally, the 5-mer peptide had a lower molecular weight and showed greater catalytic activity than other small mimics [6,12]. Based on the unique advantages of the 5-mer peptide, we constructed Hepatoma 22 (H22) cells, a commonly used murine transplanted tumor cell line that is widely applied to generate the mouse tumor models, in order to evaluate the anticancer effects of 5P in liver cancer in vitro and in vivo. Our findings provided important insights into the application of this peptide in the treatment of liver cancer.

## 2. Results

### 2.1. Kinetics of 5P

**Figure 1:**
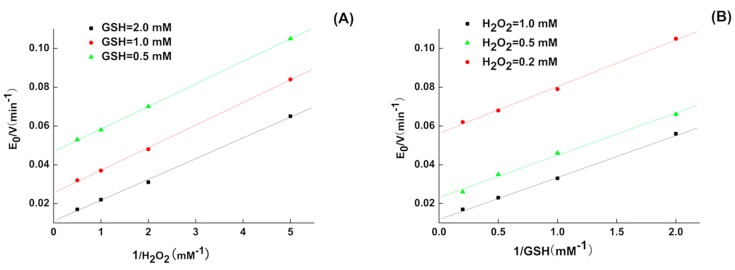
Double-Reciprocal plots for the reduction of H_2_O_2_ by GSH catalyzed by 5P. (**A**) [E_0_]/*v*_0_ versus 1/[H_2_O_2_] (mM^−1^) at [GSH] = 0.5 mM (▲), 1.0 mM (●), 2.0 mM (■); (**B**) [E_0_]/*v*_0_ versus 1/[GSH] (mM^−1^) at [H_2_O_2_] = 0.5 mM (▲), 0.2 mM (●), 1.0 mM (■).

The activity and steady-state kinetics of 5P were observed for substrates H_2_O_2_ and GSH. The activity of 5P was 10.9 U/μmol, and the relevant steady-state equation (Equation (1)) for the GPx mimic reaction was as follows:

(1) ν0[E]0=kmax[GSH]·[H2O2]Km,H2O2[GSH]+Km,GSH[H2O2]+[GSH]·[H2O2]

where *v*_0_ is the initial reaction rate, [E]_0_ is the initial enzyme mimic concentration, *k*_max_ is a pseudo-first order rate constant, and *K*_H_2_O_2__ and *K*_GSH_ are the Michaelis-Menten constants (*K*_m_) for H_2_O_2_ and GSH, respectively. From Table 1, we could see that 5P had the ability to combine with the substrate GSH (*K*_m,GSH_ = 9.4 mM), which may explain why that 5P had good catalytic capacity. The second order-rate constants (*k*_max_/*K*_m,H_2_O_2__) was 8.54 × 10^4^ M^−1^ min^−1^ and there was no significant difference with CysSeSeCys (*k*_max_/*K*_m,H_2_O_2__ = 5.3 × 10^4^ M^−1^ min^−1^) reported by B.G. Singh [13]; however, the *k*_max_/*K*_m,GSH_ (3.5 × 10^4^ M^−1^ min^−1^) was higher than CysSeSeCys (*k*_max_/*K*_m,GSH_ = 2.3 × 10^2^ M^−1^ min^−1^), suggesting that 5P may have better catalytic efficiency for GSH. Double reciprocal plots of the initial velocity versus the concentration of substrates gave a family of parallel lines (Figure 1), indicating that the reaction mechanism was a ping-pong mechanism analogous to those of the natural GPxs [14]. The affinity with both substrates was higher than that of the other substrates alone.

### 2.2. Inhibition of H22 Cells by 5P In Vitro

The growth inhibition rate is shown in Table 2. A 5P concentration of 10–25 μM inhibited H22 cell proliferation compared with the negative control. After culturing for 48 h, 5P (10 μM) had certain inhibitory effects on H22 cell proliferation; meanwhile, there were significant differences compared with the negative control.

According to the OD values determined by the 3-(4,5-dimethylthiazol-2-yl)-2-5-diphenyltetrazolium bromide (MTT) method, we obtained the linear regression equation of 5P for the tumor inhibition rate (Equation (2)), as follows:

*y* = 2.795*x* − 4.815 (2)

The half maximal inhibitory concentration (IC_50_) of 5P was 18.31 μM. These results suggested that 5P significantly suppressed the proliferation of H22 cells.

### 2.3. Effects of 5P on Apoptosis

**Figure 2:**
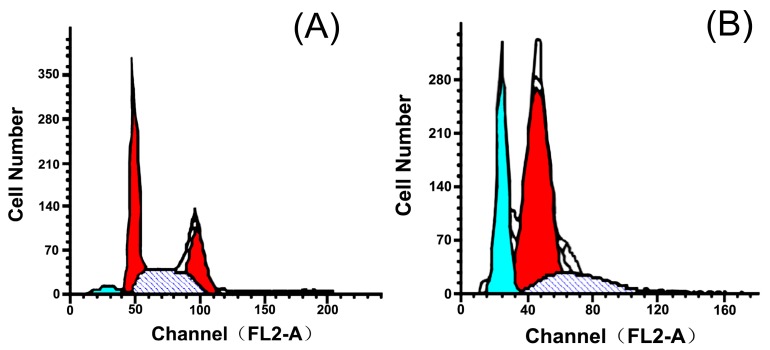
DNA cell cycle analysis in control H22 cells (**A**) and inhibitory effects of 5P (20 μM, 48 h) on H22 apoptosis (**B**). (the cyan part represents the G_o_/G_1_ phase, the red part represents the G_2_/M phase and the dark part represents the S phase).

At 48 h after treatment, 5P (20 μM) significantly blocked proliferation and accelerated apoptosis in H22 cells. The apoptosis index (AI) reached 27.51% (Table 3), and cell cycle analysis showed that 5P blocked the cell cycle in G_0_/G_1_ phase and reduced accumulation of cells in the G_2_/M and S phases (Figure 2).

### 2.4. Effects of 5P on Apoptosis-Related Genes

In order to observe changes in the transcription of apoptotic genes, we used reverse transcription polymerase chain reaction (RT-PCR). The results showed that, compared with the negative control group, 20 μM 5P reduced the expression of anti-apoptotic *Bcl-2* and upregulated pro-apoptotic *Bax* in H22 cells.

**Figure 3:**
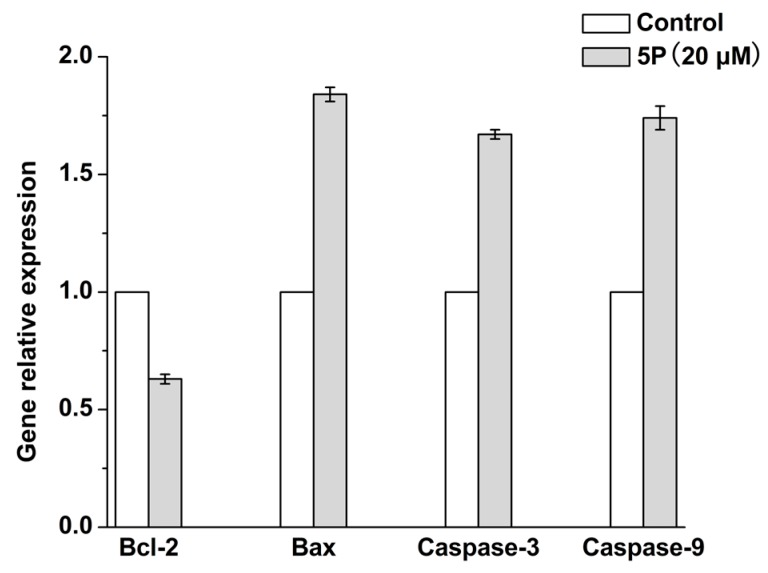
Relative expression of *Bcl-2*, *Bax*, caspase-3, and caspase-9 genes.

Caspases activate apoptosis; caspase-9 can cleave caspase-3 and other caspases [15] to activate the effector phase of apoptosis and cause damage to the cells. As shown in Figure 3, we found that 5P altered the expression of apoptosis-related genes in H22 cells, including *Bax*, caspase-9, and caspase-3. Thus, 5P activated apoptosis by enhancing the expression of caspase-9 and caspase-3.

### 2.5. In Vivo Tumor Growth Model

By observing the living conditions of mice, we determined parameters of normal body weight gain and condition. Compared with the normal group, the tumor group showed increased weight, and the cyclophosphamide (CTX)-treated group showed decreased weight. In contrast, mice in the 5P group showed increased body weight compared with the normal group (Table 4).

After continuous dosing for 10 days, CTX significantly blocked tumor growth. The tumor inhibition ratio reached 60.94%. Compared with the tumor control group, 5P clearly inhibited tumor growth, with inhibition ratios of 46.00% and 34.86% (Table 5).

**Figure 4:**
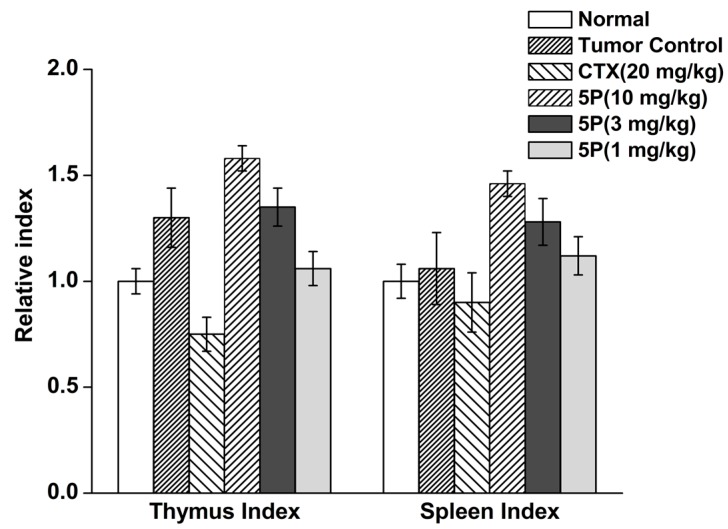
The effects of 5P on tumor growth in H22-bearing mice.

After continuous dosing for 10 days, CTX decreased the thymus and spleen indexes in H22 tumor-bearing mice. At a dose of 20 mg/kg, 5P significantly increased the thymus and spleen indexes in H22 tumor-bearing mice. Other doses of 5P did not affect the indexes compared with the tumor control group (Figure 4).

**Figure 5:**
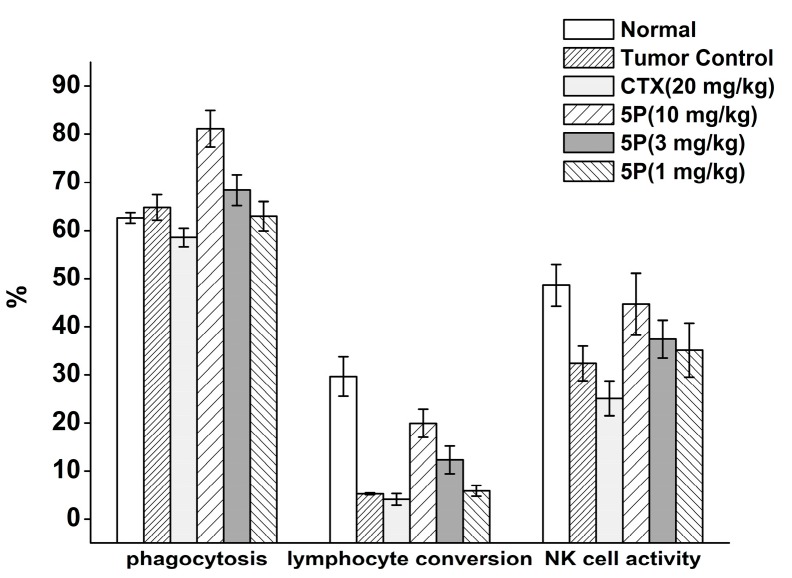
Effects of 5P on phagocytosis, lymphocyte conversion, and NK cell activity in H22 tumor-bearing mice.

Compared with the normal group, the CTX group showed significant decreases in phagocytosis, lymphocyte conversion, and natural killer (NK) cell activity. In contrast, phagocytosis, lymphocyte conversion, and NK cell activity were increased in the 5P group, with increased phagocytosis as the dose increased (Figure 5).

**Figure 6:**
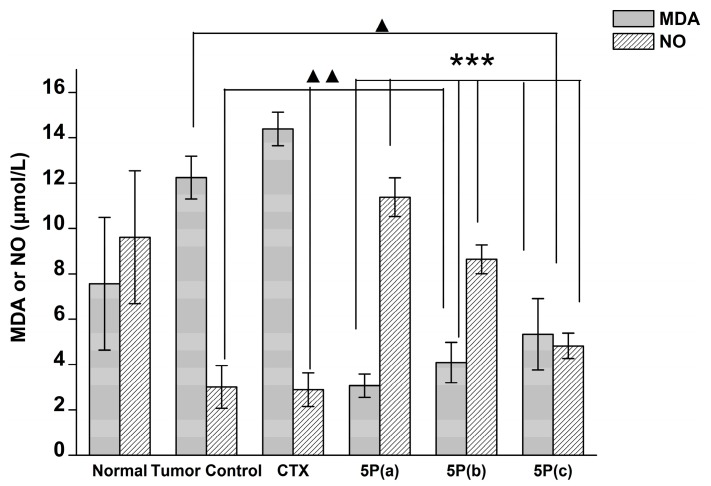
Effects of 5P on malondialdehyde (MDA) and nitric oxide (NO) contents in blood plasma of H22 tumor-bearing mice. 5P(a): 10 mg/kg, 5P(b): 3 mg/kg, 5P(c): 1 mg/kg. *** *p* < 0.001 versus the tumor control group; ^▲▲^ *p* < 0.001 versus the normal group; ^▲^ *p* < 0.05 versus the normal group.

Compared with the normal control group, the tumor control group showed significantly decreased plasma nitric oxide (NO) levels, suggesting that tumor formation could reduce NO content in mice. CTX reduced the content of NO in the plasma of tumor-bearing mice, but no significant differences were observed. The 5P-treated group showed significantly increased NO contents compared with the tumor control group (Figure 6).

**Figure 7:**
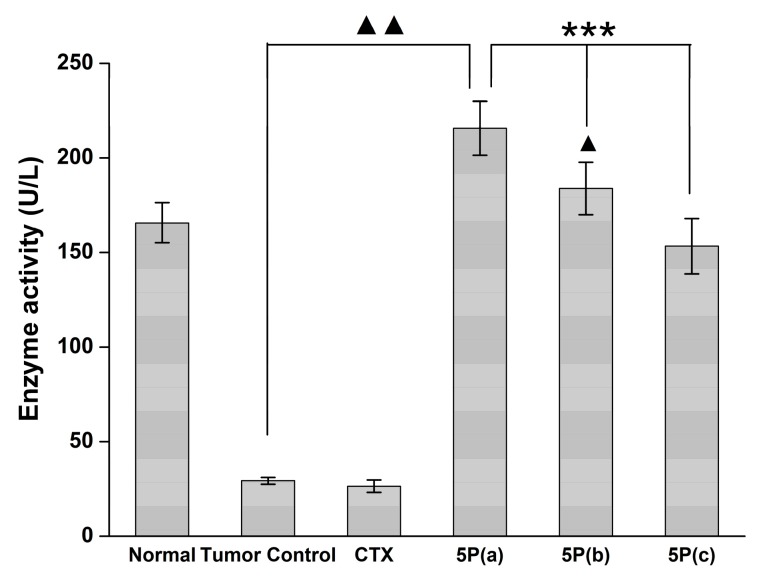
Effects of 5P on GPx activity in the blood plasma of H22 tumor-bearing mice. 5P(a): 10 mg/kg, 5P(b): 3 mg/kg, 5P(c): 1 mg/kg. *** *p* < 0.001 versus the tumor control group; ^▲^ *p* < 0.05 versus the normal group; ^▲▲^ *p* < 0.001 versus the normal group.

Notably, we found that different doses of 5P significantly increase tumor GPx activity in mouse plasma compared with the tumor control group (*p* < 0.001; Figure 7).

## 3. Discussion

GPx has strong antioxidant effects, conferring it with great medicinal potential. However, some analogs are macromolecular substances with molecular weights of more than 30 kDa, making them impractical for use as drugs. Accordingly, it is necessary to identify or synthesize compounds with lower molecular weights. Luo et al. used chemical synthesis to obtain 2-TeCD, which has high GPx activity [16]. Additionally, Sun et al. used phage display peptide library technology to obtain a 15-mer peptide (15P) and then applied computer-aided simulation of a natural GPx catalytic environment; this, combined with chemical mutation methods, resulted in successful synthesis of 15P [12]. However, the chemical mutation method has some disadvantages, such as high cost and product loss. Li et al. constructed a novel selenocysteine-containing 7-mer peptide for which the GPx activity was 13 U/µmol; this peptide showed protective effects against hepatic I-R injury [6]. In our study, we designed a 5-mer peptide (5P) and developed an inexpensive method to obtain high yields. We then used solid phase peptide synthesis and selenium-based generation of cysteine derivatives to obtain 5P directly, which could further simplify the operation, reduce the synthetic cost, and facilitate applications in drug development. The characteristic of ping-pong mechanism is that double-reciprocal plots of the initial velocity versus substrate concentration yielded a series of characteristic parallel lines for both substrates [14]. Therefore, the catalytic mechanism of 5P was consistent with that of endogenous GPx.

Liver cancer is a serious disease that threatens human health. Several studies have demonstrated that GPx has anticancer activities in liver cancer [10,17,18]. In this study, we evaluated the anticancer effects of 5P in H22 cells in vitro and in a mouse model in vivo. The results indicated that 5P could effectively inhibit the growth of H22 cells in vitro [19]. Based on our understanding of the mechanisms of 5P, we expected that this compound inhibited tumor cell growth in vitro as follows. During proliferation, tumor cells produce free radicals, which are harmful to tumor cells. 5P, as a small peptide with good catalytic activity, could enter the cell rapidly to affect protein expression levels. These changes in protein expression would affect cell cycle regulation, which alters cell division, similar to the effects of anticancer drugs, which induce cell cycle arrest and apoptosis in cancer cells [19]. In addition, 5P could cause cell cycle arrest in G_0_/G_1_ and reduce the distribution of cells in the S phase, thereby inhibiting the proliferative activity of tumor cells. *Bcl-2* and *Bax* genes regulate apoptosis; *Bcl-2* promotes cell survival, whereas *Bax* inhibits cell survival. In this study, we found that 5P could reduce the expression of *Bcl-2*, while increasing the expression of *Bax*. Most anticancer drugs induce apoptosis in cancer cells via activation of the cytochrome C/caspase 9 pathway or by affecting the mitochondrial membrane [20]. These results indicated that 5P could activate apoptosis by increasing the expression of caspase-9 and caspase-3.

To evaluate the effects of 5P on liver cancer growth, we used a mouse model in which CTX was applied as a control. CTX is an anticancer chemotherapeutic agent that is widely used in the treatment of various types of human cancers, such as hepatocellular carcinoma, medulloblastoma, and carcinomas of the breast, lung, and cervix [21]. However, a high dosage of CTX often leads to severe side effects, which greatly affect the quality of life of patients [22,23]. By detection of some indexes of immunity, we found that the 5P had significant effects on improvement of immunity compared with CTX. Moreover, some studies have suggested that antioxidants and immune function are both essential for cancer prevention [24]. Based on the results of 5P treatment in H22 tumor-bearing mice, we concluded that 5P could reduce the damage produced caused by free radicals and protect normal tissues in H22 tumor-bearing mice through the following mechanisms. First, the decrease in immunity is one of the most important factors affecting tumor occurrence. The thymus and spleen are the main immune organs and can be examined as an index of the effects of drugs on immunity [25,26]. 5P improved these indexes, enhancing their antitumor effects compared with those of the other treatment groups. Moreover, macrophages, as important nonspecific immune cells, can release NO, which is essential for the cytotoxic effects of macrophages against tumors because of its ability to induce iron release from cancer cells [27,28]. In this study, we found that 5P could improve phagocytosis by macrophages and increase NO content. Thus, we inferred that 5P could improve nonspecific immunity and the level of NO to enhance its antitumor effects. Cellular immunity is an immune response involving T lymphocytes, which are critical for tumor immunity. NK cells, which are part of the first-line antitumor response, are crucial in immune regulation [29,30]. Our findings demonstrated that 5P increase the proliferation of T lymphocytes, resulting in clearance of tumor cells. Thus, this GPx mimic showed improved tumor-killing capacity.

Lipid peroxidation is triggered by a series of reactions caused by free radicals. Malondialdehyde (MDA) is the ultimate product of lipid peroxidation, and GPx has the ability to reduce free radicals produced by tumor cells. From our results, we found that 5P improved the activity of plasma GPx, resulting in a reduction in free radicals, including MDA.

In summary, we found that 5P could overcome many of the limitations of traditional anticancer drugs. Moreover, 5P showed positive effects on the organism and could be used at low doses to inhibit tumor growth.

## 4. Materials and Methods

### 4.1. Ethics

Procedures involving animals and their care were conducted in accordance with the NIH guidelines (NIH Pub. No. 85-23, revised 1996) and approved by the Animal Ethics Committee of Jilin University.

### 4.2. Materials

GSH, glutathione reductase, *t*-BuOOH, and nicotinamide adenine dinucleotide phosphate (NADPH) were obtained from Sigma (St. Louis, MO, USA). Fetal bovine serum was obtained from HyClone Inc. (Logan, UT, USA). RPMI-1640 was from Gibco (BRL Ltd., Paisley, Scotland). MTT was purchased from Chemicon (Temecula, CA, USA). Dimethyl sulfoxide was obtained from Ameresco (Solon, OH, USA), and CTX was purchased from Hualian Co., Ltd. (Shanghai, China). *Bcl-2*, *Bax*, caspase-3, caspase-9, and diaminobenzidine (DAB) kits were purchased from BOSTER (Wuhan, China). The NO assay kit and MDA assay kit (thiobarbituric acid (TBA) method) were obtained from Nanjing Jiancheng Bioengineering Institute (Nanjing, China). H22 cells were provided by the Cancer Hospital of Jilin Province (Changchun, China). All other materials were of analytical grade and were obtained from Beijing Chemical Plant (Beijing, China).

The HPLC-2695 instrument was obtained from Waters (Milford, MA, USA), and liquid chromatography tandem mass spectrometry (Q-Trap; TQ001361/LC: 81247; AB SCIEX, Concord, ON, Canada) was used. The semi-automatic analyzer was from Antai Inc. (Shanghai, China), and the UV1700 instrument was from Shimadzu (Kyoto, Japan). The ELx808 microplate reader was from BioTek Instruments (Winooski, VT, USA). The FACScan instrument was from Becton Dickinson (Franklin Lakes, NJ, USA), and the Model AG 2331PCR instrument was from Eppendorf (Hamburg, Germany). We used a Model 680 Microplate Reader from Bio-Rad (Hercules, CA, USA).

### 4.3. Synthesis of 5P

We compounded the Fmoc-Sec (PMB)-OH with reference to the methods by Koide [30]. The GPx mimic was obtained by solid phase peptide synthesis [31]. The product was purified by reverse phase high-performance liquid chromatography (supplementary materials) and the yield was 21 mg (32.3%). The chemical state and percentage of selenium were measured by X-ray photoelectron spectroscopy (XPS) analysis in a Vacuum Generator (VG) ESCALAB Mk II spectrometer (VG Scientific, East Grinstead, UK) with an Mg Kα (1253.6 eV) achromatic X-ray source. The Se_3d_ electronic-binding energy of 5P is 54.8 eV, which approaches the binding energy of SeCys (55.1 eV), indicating that the selenium in 5P is present in the form of −1 valence (diselenium bridge, -Se-Se-). The experiment also gave a C/Se ratio of 20.7:1 (calculated 21:1), indicating that there was 2 mol of selenium per mol of [Arg-Gly-Sec-Arg-Asn]_2_. Therefore, 5P existed in the dimer form [Arg-Gly-Sec-Arg-Asn]_2_. Matrix-assisted laser desorption ionization time-of-flight mass spectrometry showed an *m*/*z* of 651.5 (calculated for [M]^+^: 650.3).

### 4.4. GPx-Like Activity and Kinetics Measurement

GPx-like antioxidant activity was assayed as previously described [32]. The reaction was carried out at 37 °C in 700 μL solution containing 50 mM (pH 7.0) potassium phosphate buffer, 1 mM ethylenediaminetetraacetic acid (EDTA), 1 mM NaN_3_, 1 mM GSH, 1 U glutathione reductase, and 10–50 μM 5P. The reaction mixture was incubated for 7 min, and 0.25 mM NADPH was then added for 3 min. The reaction was initiated by addition of 0.5 mM H_2_O_2_. The activity was determined by the decrease in NADPH absorption at 340 nm (εNADPH = 6220 M^−1^ cm^−1^). The background absorption of the noncatalytic reaction was measured without mimic and was subtracted. The activity unit of the enzyme mimic was defined as the amount of enzyme mimic that utilizes 1 μmol NADPH per min.

5P kinetics were analyzed using an assay similar to that for native GPx [33]. Initial reduction rates of H_2_O_2_ by GSH were determined by observing the change in NADPH absorption at 340 nm at 37 °C and pH 7.0, varying one substrate concentration while another remained fixed. All kinetic experiments were performed at 37 °C in 700 μL reaction solution containing 0.5–3.0 mM GSH, 0.5–2.0 mM H_2_O_2_, 50 mM potassium phosphate buffer (pH 7.0), 1 mM EDTA, 0.25 mM NAPDH, 1 U GSH reductase, and 5 μM 5P. Background absorption of the noncatalytic reaction was measured without mimic and was subtracted from the total absorption with the mimic. Kinetic data were analyzed by double-reciprocal plotting.

### 4.5. Cell Viability Assay

Cell viability was assessed using the MTT method. Following treatment of H22 cells (2.6 × 10^3^ cells/well) with various concentrations of 5P in 96-well plates for 24 h, 20 μL/well MTT (5 mg/mL) solution was added. After a 4-h incubation at 37 °C, the formazan crystals were solubilized with 150 μL/well DMSO for 10 min. The absorbance values of the solution in each well were measured at 570 nm.

### 4.6. Apoptosis Assay

Apoptosis was assessed using flow cytometry analyses with propidium iodide (PI) staining. Cells (1.4 × 10^6^) were seeded in six-well plates grown at 37 °C in a humidified incubator with 5% CO_2_ for 1 day and were then treated with 20 μM 5P. After 2 days, cells were centrifuged at 1000 rpm for 7 min, and the supernatant was collected, washed with phosphate-buffered saline, and treated with 2 mL of 70% alcohol. The next day, the samples were washed with PBS once, and the supernatant was removed. Fixed cells were resuspended in 500 μL PBS, and 100 μL RNase (10 mg/mL) and PI (10 mg/mL) were added. Cells were then incubated at 4 °C in the dark for 20 min and analyzed using a FACSCalibur (Becton Dickinson, Franklin Lakes, NJ, USA) with Cell Quest Software (Cell Quest pro, Becton Dickinson, Franklin Lakes, NJ, USA) [34].

### 4.7. RT-PCR Analysis

Total RNA was isolated from H22 cells treated with 20 μM 5P. Quantitative real-time PCR was performed to assess the expression of *Bcl-2*, *Bax*, caspase-3, and caspase-9 genes. The following primers were used: *Bcl-2* (forward: 5′-CCCCTTCATCCAAGAATGC-3′ and reverse: 5′-TTCCACAAAGGCATCCCAG-3′), *Bax* (forward: 5′-CCACCAGCTCTGAACAGTTCA-3′ and reverse: 5′-TGAGGACTCCAGCCACAAAG-3′), caspase-3 (forward: 5′-TTAGTGTCCTGAGGTGCGGA-3′ and reverse: 5′-GCGCGTACAGTTTCAGCAT-3′), and caspase-9 (forward: 5′-GGCTCTGGCTTCATTCTTG-3′ and reverse: 5′-CTCTCGATGGACACAGAGCAT-3′).

The measurement was performed using SYBR Green assays and an ABI 7500 System (Applied Biosystems, Foster City, CA, USA). The results were calculated using the following the formula: [2_−(CT target − CTGAPDH)_] × 100%.

### 4.8. In Vivo Tumor Growth Model

We used H22 tumor-bearing mice in this study. The study had six groups, as follows: 5P high-dose group (10 mg/kg), 5P middle-dose group (3 mg/kg), 5P low-dose group (1 mg/kg), positive control (CTX, 20 mg/kg), tumor control group (normal saline), and normal group (normal saline). All groups received treatment by intraperitoneal injection. The treatment was given at 09:00 every day and dosing was continued for 10 days (10 mg/kg). For our assays, each group was killed under anesthesia using avertin. According to the methods of Nagata [35], we evaluated the phagocytosis of phagocytes. On day 8, the mice were treated with soluble starch by intraperitoneal injection. For cellular immunity, we used MTT colorimetric assays to detect the multiplication capacity of T lymphocytes. By calculating the mean OD values of the experimental and control groups, we could obtain the conversion ratios of the T lymphocytes. Natural killer (NK) cells are the first line of antitumor response. Based on the literature [36], we evaluated the activity of NK cells. The concentration of splenocytes was adjusted to 5 × 10^6^/mL, and that of YAC-1 cells was 1 × 10^5^/mL.

### 4.9. Blood MDA, NO, and GPX Activity

Blood samples were obtained via the orbital vein after 6 h. MDA was evaluated using TBA assays. NO levels were assessed using the nitric acid reductase method. We could calculate the content (μM) of NO using the assay kit. Measurement of GPx activity was described above.

### 4.10. Tumor Weights, Thymus Index, and Spleen Index

Under aseptic conditions, the thymus and spleen were dissected. The tumor control rate was calculated as follows: (1 − experimental tumor weight/control group tumor weight) × 100%; the thymus index was calculated as follows: thymus weight (mg)/weight (g); and the spleen index was calculated as spleen weight (mg)/weight (g).

## Figures and Tables

**Table 1:** Kinetic parameters of the 5P-catalyzed reduction of H_2_O_2_ by glutathione (GSH).

GPX Mimic	*k*_max_ (min^−1^)	*K*_m,GSH_ (mM)	*K*_m,H_2_O_2__ (mM)	*k*_max_/*K*_m,GSH_ (M^−1^ min^−1^)	*k*_max_/*K*_m,H_2_O_2__ (M^−1^ min^−1^)
5P	333.2 ± 8	9.4 ± 0.28	3.9 ± 0.12	3.5 × 10^4^	8.54 × 10^4^

**Table 2:** Inhibition of H22 cell proliferation by 5P in vitro detected by 3-(4,5-dimethylthiazol-2-yl)-2-5-diphenyltetrazolium bromide (MTT) analysis.

Group	Dose (μM)	Absorption Value (OD 570 nm)	Inhibition Ratio (%)
control	-	0.336 ± 0.026	-
5P	5	0.353 ± 0.019	-
5P	10	0.266 ± 0.025 **	20.84
5P	15	0.172 ± 0.022 ***	48.81
5P	20	0.150 ± 0.008 ***	55.36
5P	25	0.139 ± 0.007 ***	58.63

** *p* < 0. 01 versus the control group; *** *p* < 0.001 versus the control group.

**Table 3:** Inhibition of H22 proliferation by 5P in vitro detected by flow cytometry analysis.

Group	Dose	G_0_/G_1_	G_2_/M	S	Apoptosis (%)
Control	-	41.33	27.30	31.37	4.52
5P	20	79.55	0.15	20.30	27.51

**Table 4:** Effects of 5P on the body weights of H22 tumor-bearing mice.

Group	Dose mg/kg	Body Weight (g) Start	Body Weight (g) End
Normal	-	34.86 ± 2.06	40.24 ± 1.67
Tumor Control	-	34.94 ± 2.39	44.19 ± 2.92 ^▲▲^
CTX	20	34.91 ± 2.56	31.99 ± 2.27 *** ^▲▲▲^
5P	10	34.53 ± 1.98	43.34 ± 3.54 ^▲^
5P	3	34.34 ± 2.55	43.69 ± 3.10 ^▲^
5P	1	34.78 ± 3.66	43.78 ± 3.90 ^▲^

*** *p* < 0.001 versus the tumor control group; ^▲^ *p* < 0.05 versus the normal group; ^▲▲^ *p* < 0.001 versus the normal group; ^▲▲▲^ *p* < 0.001 versus the normal group.

**Table 5:** The effect of 5P on tumor growth in H22 bearing mice.

Group	Dose (mg/kg)	Tumor Weight (g)	Tumor Inhibition Ratio (%)
Normal	-	-	-
Tumor Control	-	1.09 ± 0.30	-
CTX	20	0.43 ± 0.16 ***	60.94
5P	10	0.59 ± 0.14 **	46
5P	3	0.71 ± 0.14 *	34.86
5P	1	0.91 ± 0.30	16.91

* *p* < 0.05 versus the tumor control group; ** *p* < 0.01 versus the tumor control group; *** *p* < 0.001 versus the tumor control group.

## Supplementary Materials

The following are available online.
