# Supplementary material for: The Small Glutathione Peroxidase Mimic 5P May Represent a New Strategy for the Treatment of Liver Cancer
Source: Molecules. 2017 Sep 8;22(9):1495. doi: 10.3390/molecules22091495 (PMC6151655; doi:10.3390/molecules22091495)
Supplement: Supplementary file 1 [file molecules-22-01495-s001.pdf]

Supporting Information

The Small Glutathione Peroxidase Mimic 5P May Represent a New Strategy for the Treatment of Liver Cancer

Juxin Yin [1,2], Bingmei Wang [1], Xuejun Zhu [1], Xiaonan Qu [1], Yi Huang [1], Shaowu Lv [1,*], Ying Mu [1,2] and Guimin Luo [1]

[1] Key Laboratory for Molecular Enzymology and Engineering of the Ministry of Education, College of Life Science, Jilin University, Changchun 130000, China; yinjuxin@163.com (J.Y.); wangbingmei@163.com (B.W.); zhuxuejun@163.com (X.Z.); quxn1077@163.com (X.Q.); huangyimzd@126.com (Y.H.); muying@zju.edu.cn (Y.M.); gmluo@163.com (G.L.)

[2] Research Center for Analytical Instrumentation, Institute of Cyber-Systems and Control, State Key Laboratory of Industrial Control Technology, Zhejiang University, Hangzhou 310000, China

* Correspondence: lvshaowu@163.com; Tel.: +86-135-0432-8331

Synthesis of 5P

(1) 0.1 mmol Fmoc-Asn(Trt)-Wang-Resin and 4 mL dimethyl formamide (DMF) were added to the column reactor, shaken at 25°C for 30 min, swollen, and dried, and the mixture was then washed six times for 3 min each.

(2) Add 4 mL 20% piperidine/N-methyl-2-pyrrolidone (NMP), shake the samples at 30°C for 30 min, and remove the resin Fmoc protection group. Then dry the samples, wash with DMF six times with shaking (3 min each time), and dry the samples.

(3) To the column reactor, add 0.3 mmol Fmoc-Arg(Pbf)-OH, 0.3 mmol (benzotriazol-1-yloxy) tris (dimethylamino) phosphonium hexafluophosphate (BOP), 0.3 mmol 1-hydroxybenzotriazole (HOBT), and 0.45 mmol 4-methylmorpholine. Next, add 4 mL DMF and shake three times at 25°C for 90 min.

(4) Repeat step 2 to remove the N-terminal Fmoc protecting group. Introduce Fmoc-Sec(PMB)-OH, Fmoc-Gly-OH, and Fmoc-Arg(Pbf)-OH sequentially to the resin.

(5) After adding the last amino acid, remove the N-terminal Fmoc protecting group, dry, wash with DMF six times with shaking (3 min each time), and dry the samples.

(6) Wash the resulting peptide-linked resin five times with anhydrous methanol for 2 min each time, and dry the mixture each time, with a final drying step in a vacuum oven at room temperature.

(7) First, add lysis solution (trifluoroacetic acid [TFA]:1,2-ethanedithiol:anisole = 38:1:1) to the dried reactor at 25°C for 120 min. Filter to remove the resin. Dry most of the TFA with nitrogen, add the concentrated solution dropwise into 40 mL ether:petroleum ether (1:2, V/V), and shock the sample. To the resin, add 4 mL lysis solution for 0.5 h and drip with ether/petroleum ether. Place the samples at 20°C for 1 h and centrifuged (5,000 g) 10min at 4°C. Pour the supernatant carefully, and dry in vacuo. Next, take the sample prepared above and dissolve in 4 mL of 10% DMSO/TFA at room temperature for 30 min. Place the reaction solution at -20°C for 1 h and centrifuge (5,000 g) at 4°C. Carefully pour the supernatant and wash the precipitate with the appropriate amount of ether. Centrifuge the sample (5,000 g) at 4°C for 10 min and dry the remaining ether in vacuo.

(8) Purify the residue by RP-HPLC: A was 0.1% aqueous TFA solution; B was 70% acetonitrile, 0.1% TFA. Perform gradient elution with B from 30% to 70% and a flow rate of 1 mL/min for 25 min. Detect samples at wavelengths of 214 and 385 nm. Collect the main components by freeze-drying. The product was obtained at a weight of 21 mg (32.3%).

(9) The purify analysis of 5P was used the HPLC. The HPLC system was equipped with an auto sampler and an UV detector using a C18 column (150 mm × 50 mm, 4.6 μm). A was 0.1% aqueous TFA solution; B was acetonitrile. Perform gradient elution with A from 20% to 50% and a flow rate of 1 mL/min for 28 min. Detect samples at wavelengths of 214 and 385 nm. The molecular weight was determined by Matrix-assisted laser desorption ionization time-of-flight mass spectrometry.

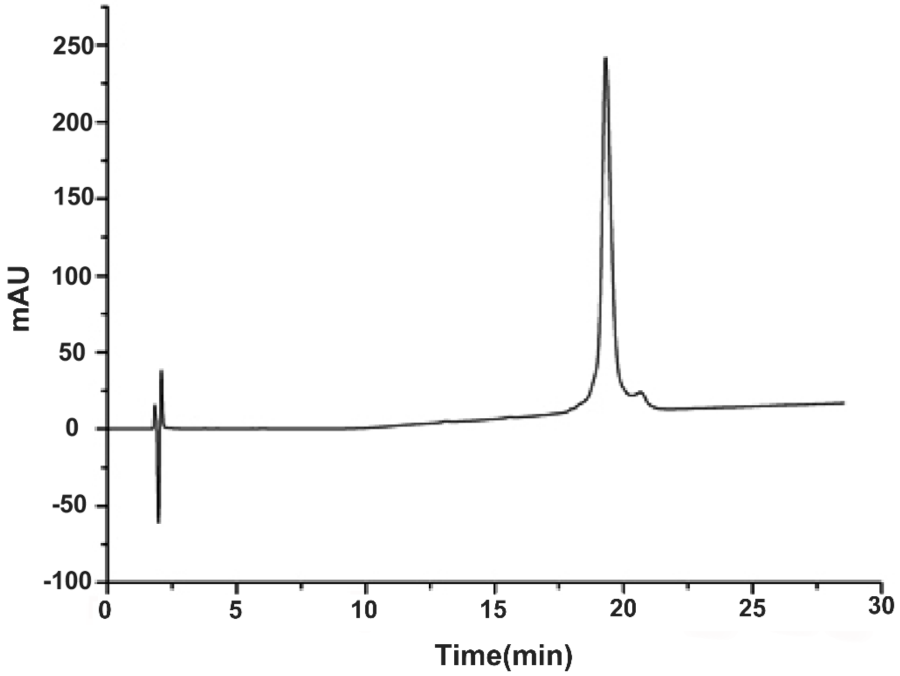

Figure S1. The purity analysis of the 5P by HPLC.

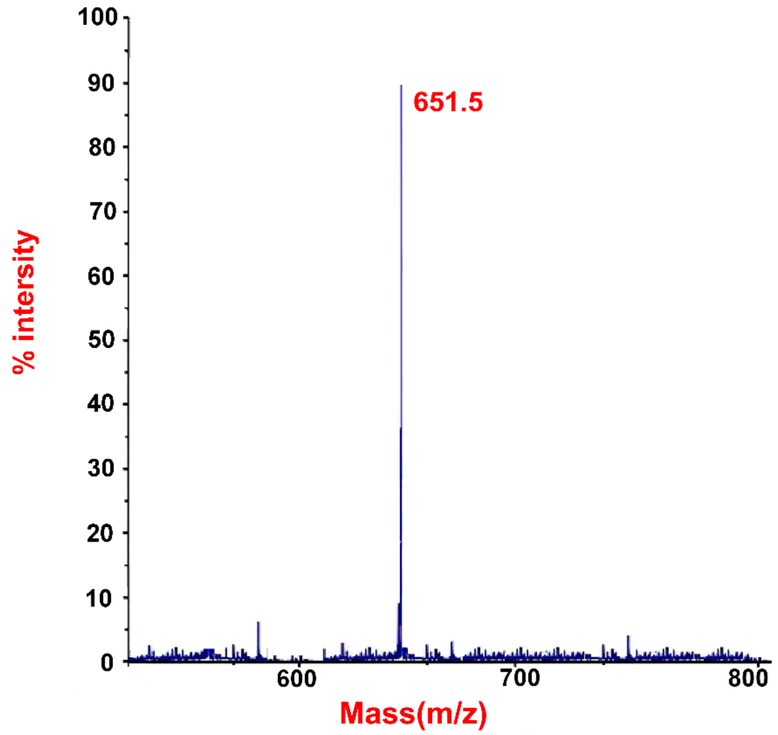

Figure S2. Identification of 5P by the Matrix-assisted laser desorption ionization time-of-flight mass spectrometry.
